# Suppression of T-Cell Proliferation by Normal Density Granulocytes Led to CD183 Downregulation and Cytokine Inhibition in T-Cells

**DOI:** 10.1155/2022/8077281

**Published:** 2022-11-16

**Authors:** Julia Westerlund, Sandra Askman, Åsa Pettersson, Thomas Hellmark, Åsa C. M. Johansson, Markus Hansson

**Affiliations:** ^1^Lund University, Department of Laboratory Medicine, Division of Hematology and Transfusion Medicine, BMC B13, 22184 Lund, Sweden; ^2^Skåne University Hospital, Department of Respiratory Medicine and Allergology, 22185 Lund, Sweden; ^3^Lund University, Skåne University Hospital, Department of Clinical Sciences Lund, Nephrology, Barngatan 2, 22185 Lund, Sweden; ^4^Skåne University Hospital, Region Skåne, Clinical Genetics and Pathology, 22185 Lund, Sweden; ^5^Skåne University Hospital, Department of Hematology, Oncology and Radiation Physics, 22185 Lund, Sweden; ^6^University of Göteborg, Sahlgrenska Academy, Institute of Medicine, Department of Internal Medicin and clinical nutrition, Bruna stråket 5, Plan 5, 41325 Göteborg, Sweden

## Abstract

Normal density granulocytes (NDGs) can suppress T-cell responses in a similar way as myeloid-derived suppressor cells (MDSCs). In this study, we tested the hypothesis that NDGs from healthy donors preferentially inhibit T helper 1 (Th1) cells and investigated the myeloid-derived suppressive effect in different T-cell populations. We found that NDG-induced suppression of T-cell proliferation was contact dependent, mediated by integrin CD11b, and dependent on NDG-production of reactive oxygen species (ROS). The suppression was rapid and occurred within the first few hours of coculture. The suppression did not influence the CD8^+^/CD4^+^ ratio indicating an equal sensitivity in these populations. We further analyzed the CD4^+^ T helper subsets and found that NDGs induced a loss of Th1 surface marker, CD183, that was unrelated to ligand-binding to CD183. In addition, we analyzed the Th1, Th2, and Th17 cytokine production and found that all cytokine groups were suppressed when T-cells were incubated with NDGs. We therefore concluded that NDGs do not preferentially suppress Th1-cells. Instead, NDGs generally suppress Th cells and cytotoxic T-cells but specifically downregulate the Th1 marker CD183.

## 1. Introduction

The neutrophil is the most common leukocyte found in the peripheral blood (60-70%) forming an important first line defense against pathogens. Neutrophils have earlier been recognized as a homogenous group with mainly one function: to find and kill invading pathogens. During the last years, neutrophils have been found to have a more diverse role in the immune system, e.g., through regulating T-cell proliferation and thereby the adaptive immune response. The neutrophil subset capable of suppression of the adaptive immune response is typically referred to as polymorphonuclear myeloid-derived suppressor cells (PMN-MDSCs) (1). Patients with different type of cancers often have increased levels of PMN-MDSCs in peripheral blood, and PMN-MDSCs are often associated with a dismal prognosis (2).

Neutrophils and PMN-MDSC are morphologically and immuno-phenotypically almost identical, and density gradient centrifugation is the preferred method to separate PMN-MDSC from neutrophils (1). Thus, PMN-MDSCs are often named low-density granulocytes (LDGs), in contrast to normal density granulocytes (NDGs) (3). Activated NDGs are also capable to suppress several T-cell functions (4–7). Aarts et al. have proposed that only mature NDGs are responsible for the suppression of T-cells, and that the suppression is mediated by NDG production of reactive oxygen species (ROS) (8).

Several mechanisms have been suggested to mediate the myeloid suppression on T-cells. ROS inhibits T-cells by lowering DNA synthesis and through alteration of T-cell receptor (TCR) signaling (9). Arginase has been shown to inhibit T-cells by depleting L-arginine from the microenvironment, which leads to downregulation of the CD3*ζ*-chain (10). In colon cancer, it has been suggested that neutrophils secrete MMP9, which in turn activates the inactive form of TGF-*β* in the microenvironment and thereby promote the inhibition of T-cells (11). There are also studies suggesting that the inhibition is contact dependent (4, 12, 13) and integrin Mac-1 (CD11b/CD18), and programmed death-ligand 1 (PD-L1) are thought to be the two main pathways (4, 12, 13). However, the inhibitory mechanisms may differ between NDGs from healthy individuals and patients.

T-cells can be divided into multiple different subsets that all display a variety of functions within the immune system. The two major subsets are the CD4^+^ T helper (Th) cells and the CD8^+^ cytotoxic T-cells. The Th cells can be further divided into Th1, Th2, and Th17 cells based on cell surface expression and cytokine production (14, 15). When it comes to cell surface expression, Th1 cells can be distinguished from the other Th subsets based on their expression of CD183 (also known as CXCR3) and their expression of CD196 (CCR6) (14). Th1 cells are CD183^+^CD196^−^ cells, whereas Th2 are CD183^−^CD196^−^, and Th17 are CD183^−^CD196^+^. Th1 cells produce, for example, interferon (IFN)-*γ* and tumor necrosis factor (TNF) and interleukin (IL)-2 and play a major role in the defense against intracellular pathogens (15, 16). Th2 cells are part of the defense against extracellular parasites and produce mainly IL-4 (15). Th17 cells are important players in the immune defense against extracellular bacteria and fungi and are the main producers of IL-17 (15).

The Th1 surface marker CD183 has four known ligands, including CXCL4 (PF4), CXCL9 (MIG), CXCL10 (IP-10), and CXCL11 (I-TAC) (17, 18). Binding of the IFN-*γ* inducible ligands CXCL9, CXCL10, or CXCL11, to CD183 promotes Th1 immune responses and Th1 migration (19) (20). CXCL4, on the other hands, seems to promote a Th2 response by promoting the production of the Th2-type cytokines (18). CXCR3 binding leads to internalization of the receptor and therefore removal from the surface of T-cells (19).

Besides becoming anergic to stimuli and lose their ability to proliferate, not much is known of how T-cells react when they encounter neutrophils. Aarts et al. has suggested that neutrophils take up pieces of T-cell membrane through trogocytosis, which lead to morphological changes and mitochondrial dysfunction (4). How different Th subsets react after neutrophil encounter has not yet been investigated.

Our group has recently shown that NDGs from healthy donors have the ability to suppress T-cell proliferation as well as IFN-*γ* production, without being preactivated (21). This inhibition was dependent on the production of reactive oxygen species (ROS). Interestingly, bone marrow NDGs from healthy donor did not suppress T-cell proliferation but IFN-*γ* production (21).

In this study, we further evaluate the mechanism by which NDGs suppress T-cell proliferation and how they affect different T-cell subsets by investigating cell surface markers and cytokine production.

## 2. Material and Methods

### 2.1. Healthy Donors

In this study, 19 healthy donors were enrolled after having signed informed consent form. Characteristics of the study population can be viewed in Supplementary Table 1. None of the donors had ongoing infections or known diseases at the point of sampling. Out of the 19 participants, 12 were female and 7 were male. The median age of the whole study population was 44.5 years of age, 47.9 for the women and 38.7 for the males. The study was approved by The Regional Ethical Review Board in Lund, Sweden, Ref No 2016/768.

### 2.2. Isolation of T-Cells

Blood was collected in 5 ml heparin tubes, whereupon the peripheral blood mononuclear cells (PBMCs) were collected after Lymphoprep™ (Axis-Shield) separation. T-cells were isolated from the PBMCs using an EasySep™ human T-cell isolation kit (Stemcell technologies) according to manufacturer's instructions. The purity of the T-cells was >97%. The 1-3% that was not T-cells was mostly debris or an occasional monocyte. In experiments where the proliferation was measured, the cells were stained with 1 *μ*M carboxyfluorescein succinimidyl ester (CFSE) (BD Horizon) for 10 min at 37°C.

### 2.3. Isolation of NDGs

NDGs were isolated from peripheral blood and collected in heparin tubes. The first step in the isolation process was Lymphoprep, followed by the lysis of RBC using 0.84% NH_4_Cl. The NDGs were then isolated using an EasySep™ human neutrophil isolation kit (Stemcell technologies) according to manufacturer's instructions. The NDGs purity was checked by flow cytometry and was >95%, and the impurities did not form any clear cell populations and was considered debris mixed with an occasional lymphocyte or monocyte.

### 2.4. T-Cell Proliferation Assay with NDGs

T-cells (100 000 cells/well) stained with CFSE were cocultured with NDGs (5 000-50 000 cells/well) for 3 days in flat-bottom 96-well plates (Eppendorf, Hamburg, Germany) coated with anti-CD3 monoclonal antibodies (1 *μ*g/ml, clone OKT-3, Invitrogen, Carlsbad, CA, United States) and anti-CD28 monoclonal antibodies (2 *μ*g/ml, clone CD28.2, Invitrogen). As a control, T-cells were added to wells pretreated with PBS to prevent activation. Culture medium used was RPMI-1640 without L-glutamine (Sigma, Malmö, Sweden) supplemented with 10% fetal calf serum (Gibco™, Thermo Fisher, Waltham, MA, United States) 10^4^ U/ml penicillin (Gibco™, Thermo Fisher), 10 ng/ml streptomycin (Gibco™, Thermo Fisher), and 2 mM L-glutamine (Gibco™, Thermo Fisher). The final volume in each well was 200 *μ*l. To evaluate how NDGs inhibit T-cell proliferation, ROS inhibitor catalase (Sigma, Malmö, Sweden), arginase inhibitor nor-NOHA (300 *μ*M, AH diagnostics, Solna, Stockholm), TGF-*β* inhibitor Galunisertib (GAL) (0.1 ug/ml, Cayman Chemicals), or anti-CD11b antibody (1 ug/ml, clone: ICRF44, eBioscience) was added to the cocultures. All substances were used in concentrations that did not affect T-cell viability. In some experiments, 1 *μ*M of N-Formylmethionyl-leucyl-phenylalanine (fMLF) was added to the cocultures to activate neutrophils. After 3 days of coculture, the supernatant was saved and stored in -80°C, and the proliferation of the T-cells was evaluated on a CytoFLEX (Beckman coulter).

### 2.5. Cytometric Bead Array (CBA)

Supernatant from the 3-day long T-cell and NDGs cocultures were thawed, diluted 1 : 10, and used in a human Th1/Th2/Th17 cytometric bead array (CBA) according to manufacturer's instructions (BD). The presence of interleukin-(IL-) 2, IL-4, IL-6, IL-10, tumor necrosis factor (TNF), interferon (IFN)-*γ*, and IL-17A was measured. The data was analyzed using FCAP array multiplex assay analysis software.

### 2.6. Time Experiment

Healthy donor T-cells (100 000 cells/well) were cultured together with NDGs (50 000 cells/well), yielding a final volume of 200 *μ*l/well. At several time-points, ranging from 0.5 to 72 h, the cells were taken out and analyzed. Viability was tested using FITC Annexin V Apoptosis Detection Kit I (BD Pharmingen) according to manufacturer's instructions and the phenotype of the T-cells was analyzed on a CytoFLEX using a T-cell antibody panel (Supplementary Table 2). All flow cytometry data were analyzed using the Kaluza software (Beckman coulter).

### 2.7. Statistical Analysis

Statistical analysis was performed using GraphPad Prism Version 9 (GraphPad Software, San Diego, CA, United States). The data was not normally distributed, therefore nonparametric tests were used. Wilcoxon matched pairs signed rank test was used to compare groups.

## 3. Results

### 3.1. The Inhibitory Effect of NDGs Is Dependent on Cell-Cell Contact and ROS Production

In a previous study from our group, we showed that NDGs have the ability to inhibit T-cell proliferation through the production of ROS (21). In this study, we further evaluate possible inhibitory mechanisms through which neutrophils inhibit T-cell proliferation. T-cells were cultured together with NDGs or with a combination of NDGs and an inhibitor. T-cell proliferation was measured after 3 days, where the effect of catalase, nor-NOHA, GAL, and anti-CD11b, was determined. As a control, T-cells and activated T-cells were cultured alone with these inhibitors, but they did not show any toxicity and did not affect proliferation (data not shown).

**Figure 1:**
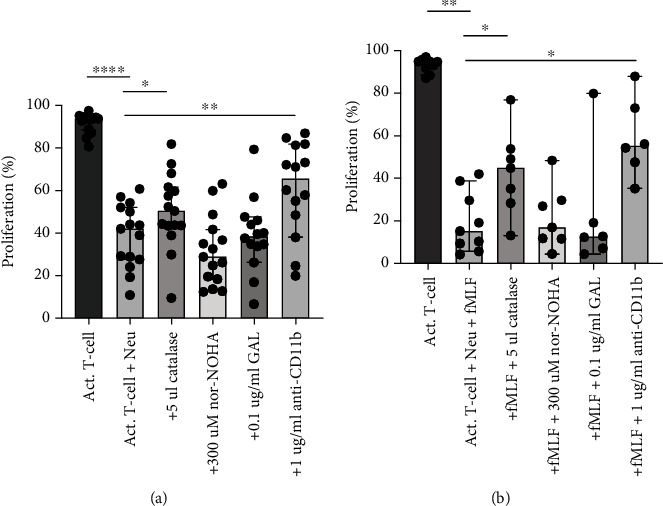
Inhibition of T-cell proliferation by NDGs and the effect of catalase, nor-NOHA, GAL, and CD11b on proliferation restoration. (a) Inhibition of T-cell proliferation by nonactivated NDGs (*n* = 15). Neutrophils suppress T-cell proliferation (*p* < 0.001), and addition of ROS inhibitor catalase protects T-cell proliferation (*p* = 0.048). The arginase inhibitor nor-NOHA did not have a protective effect and neither did TGF-*β* inhibitor GAL. Addition of anti-CD11b antibody protected T-cell proliferation (*p* = 0.0040). (b) Inhibition of T-cell proliferation by fMLF activated NDGs (*n* = 9). fMLF activated NDGs inhibit T-cell proliferation (*p* = 0.0039), and the proliferation was improved by catalase (*p* = 0.016) and anti-CD11b (*p* = 0.031). nor-NOHA and GAL did not improve proliferation. Graphs indicate median with 95% CI, and statistical significance was tested using Wilcoxon matched pairs signed rank test.

In line with our previously published data, nonactivated NDGs inhibited T-cell proliferation (*p* < 0.0001) with an inhibition range from 33% to 78% (Figure 1(a)). Catalase, a ROS inhibitor, protected T-cell proliferation partially (*p* = 0.048), while the addition of arginase inhibitor nor-NOHA did not affect proliferation. In addition, blocking of CD11b by anti-CD11b antibody restored T-cell proliferation partially (*p* = 0.0040) by blocking cell-cell contact between NDGs and T-cells (Figure 1(a)). Supernatant transfer from cultured NGDs to stimulated T-cells did not affect proliferation (data not shown). Moreover, to investigate if the inhibition of T-cell proliferation was mediated by TGF-*β* activated matrix metalloprotease, the TGF-*β* inhibitor GAL was added to the culture. GAL did not protect the proliferation.

NDGs activated with fMLF showed similar patterns of inhibition and restoration (Figure 1(b)). The fMLF activated NDGs inhibited T-cell proliferation (*p* = 0.0039) with an inhibition ranging from 53% to 93%. In all cases, fMLF activated NDGs have stronger inhibitors than those in the nonactivated NDGs from the same donor (data not shown). Both catalase (*p* = 0.016) and anti-CD11b (*p* = 0.031) restored the proliferation, while nor-NOHA and GAL did not have this ability.

These data indicate that both nonactivated and activated NDGs are strong T-cell inhibitors, and that the inhibition is mediated by ROS production and cell-cell contact but not through arginase and TGF-*β* activation.

### 3.2. NDG-Induced T-Cell Inhibition Is Rapid and Begin within the First Few Hours of Coculture

**Figure 2:**
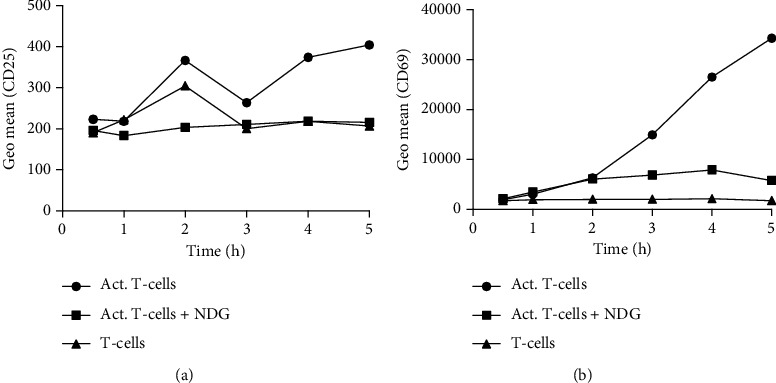
CD25 and CD69 expression on CD3^+^ T-cells during 0.5–5 hours coculture with NDG. (a) The geometric mean of CD25 on T-cells, activated T-cells, and activated T-cells cocultured with NDGs for 0.5–5 h. (b) The geometric mean of CD69 on T-cells, activated T-cells, and activated T-cells cocultured with NDGs for 0.5–5 h. Graphs show the median of 5 experiments.

CD25 (IL-2 receptor *α*-chain) and CD69 (transmembrane C-type lectin) are common T-cell markers that can be used to monitor T-cell activation. CD69 is a marker of early T-cell activation and involved in T-cell proliferation, while CD25 is a slower activation marker. To investigate how fast T-cells respond to NDGs, CD25 and CD69 were measured on nonactivated T-cells, activated T-cells, and activated T-cells in coculture with NDGs at six time-points between 0.5 and 5 hours of culture. The cells were gated according to Supplementary Figure 1, and the expression of CD25 and CD69 were analyzed (Figure 2). T-cells and NDGs were viable throughout the experiment.

Both CD25 and CD69 increased on the activated T-cells during the first 5 hours of coculture (Figure 2). CD69 increased more than CD25, which is expected since CD69 is a marker of earlier T-cell activation. NDGs inhibited the accumulation of CD25 and CD69 on the surface of T-cells, indicating that NDG inhibition of T-cells is rapid and occurs within the first few hours of coculture.

### 3.3. NDGs Downregulate the Th1 Cell Surface Marker CD183 on T-Cells

Reduced IFN-*γ* production is commonly used to show MDSC-induced inhibition of cytokine production in T-cells. Since IFN-*γ* is mainly produced by CD8^+^ cells and Th1 cells, we hypothesized that NDGs have a differential inhibitory effect on Th-cell subsets. To test this hypothesis, T-cells were cultured with NDGs, at a 1 : 2 ratio, for 0.5–72 h, making it possible to track the levels of CD4^+^ and CD8^+^ T-cells over time. The CD4^+^ cells were gated into Th1, Th2, and Th17 cells based on their expression of CD183 (CXCR3) and CD196 (CCR6). Th1 cells were defined as CD3^+^CD4^+^CD183^+^CD196^−^ cells, Th2 cells as CD3^+^CD4^+^CD183^−^CD196^−^, and Th17 cells as CD3^+^CD4^+^CD183^−^CD196^+^. Gating strategies are shown in Supplementary Figure 1.

**Figure 3:**
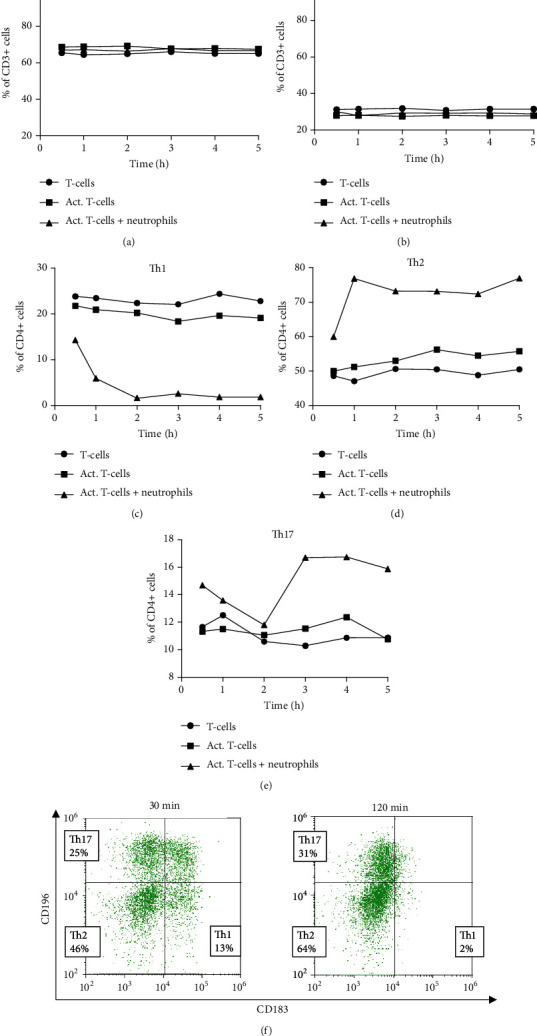
NDGs downregulate the Th1 marker CD183 on T-cells. T-cells were cultured together with NDGs for 0.5–5 hours, and T-cell subsets were evaluated at different time-points. NDGs did not change the percentage of (a) CD4^+^ cells and (b) CD8^+^ cells. However, the percentage of (c) Th1 cells decreased, and the percentage of (d) Th2 and (e) Th17 cells increased. (f) Subsets of T helper cells were distinguished based on their expression of CD196 and CD183. Graphs (a)–(e) show the median of 5 experiments.

We found that incubation with NDGs did not change the ratio between CD4^+^ and CD8^+^ T-cell subsets. However, Th1 cells rapidly decreased already after 30 min and did not recover over the 5 h period (Figures 3(a)–3(c)). In contrast to Th1 cells, both Th2 and Th17 subsets increased (Figures 3(d)–3(e)). In the coculture samples, the CD183 expression declined at 30 min and was almost completely lost after 120 min (Figure 3(f)). No decline of CD183 was observed in controls incubated without NDGs (Figure 3(f)).

When the activated T-cells proliferate, they increase in size and autofluorescence, making it impossible to accurately track the different T helper subsets after 5 h. However, this does not occur when T-cells are cultured without stimulation as they do not proliferate, making it possible to observe the different subsets even after 5 h of culture. When comparing the nonactivated T-cells cultured with and without NGDs, the decline of CD183 was not as rapid as for activated T-cells but was observed after 24 h (data not shown).

This indicates that NDGs suppress CD183 expression on T-cells overall, not just on activated T-cells. The observed decline of CD183 on Th1 cells may not represent a true Th1 decline but could be a decline in overall CD183 expression.

### 3.4. NDGs Suppress Cytokine Production in T Helper Subsets

To further test whether NDGs promote the formation of Th2 and Th17 subsets, we performed a cytometric bead array (CBA). The CBA measured seven different Th1, Th2, and Th17 specific cytokines, including IL-2, IL-4, IL-6, IL-10, IL-17A, IFN-*γ*, and TNF, in the supernatant from 3-day cocultures. IL-2, TNF, and IFN-*γ* are Th1 cytokines, while IL-4, IL-6, and IL-10 are Th2 cytokines, and IL-17A is a Th17 cytokine.

**Figure 4:**
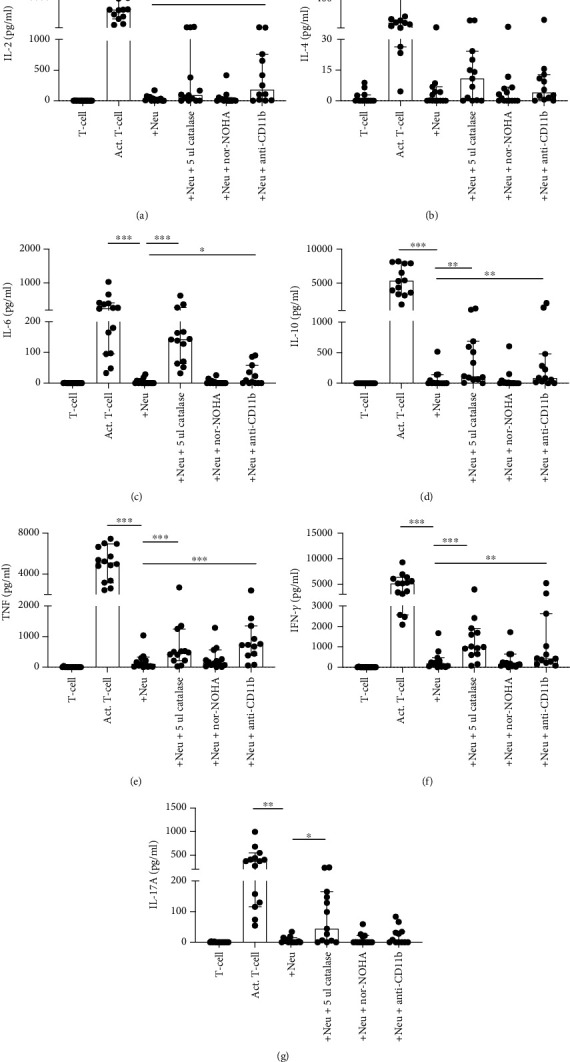
NDGs inhibit the cytokine production of Th cells. After 3 days of coculture together with the inhibitors catalase, nor-NOHA, and anti-CD11b, the supernatant was saved and used in CBA to measure the presence of (a) IL-2, (b) IL-4, (c) IL-6, (d) IL-10, (e) TNF, (f) IFN-g, and (f) IL-17A. Graphs show median with 95% CI (*n* = 14).

**Figure 5:**
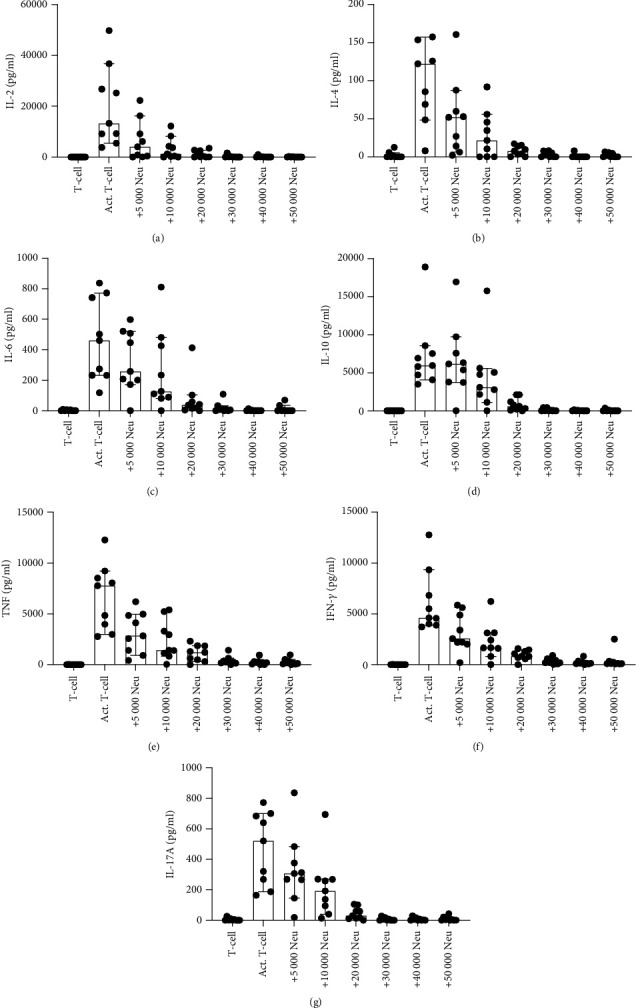
NDGs suppress cytokine production of Th cells in a dose-dependent manner. T-cells (100 000/well) were cocultured with different amounts of NDGs (5000, 10 000, 20 000, 30 000, 40 000, or 50 000/well). After 3 days of coculture, the supernatant was saved and used in a CBA to measure the presence of (a) IL-2, (b) IL-4, (c) IL-6, (d) IL-10, (e) TNF, (f) IFN-g, and (f) IL-17A. Graphs show median with 95% CI (*n* = 9).

We found that the presence of NDGs inhibited the production of all the measured T-cell cytokines (*p* = 0.0001) (Figure 4), in a dose-dependent manner (Figure 5). The addition of the ROS inhibitor catalase in the cocultures partly rescued the production of all cytokines, including IL-2 (*p* = 0.020), IL-4 (*p* = 0.0059), IL-6 (*p* = 0.0002), IL-10 (*p* = 0.0024), TNF (*p* = 0.0002), IFN-*γ* (*p* = 0.0007), and IL-17A (*p* = 0.0137). The arginase inhibitor nor-NOHA did not protect cytokine production, while the presence of anti-CD11b increased the production of IL-2 (*p* = 0.0010), IL-4 (*p* = 0.020), IL-6 (*p* = 0.023), IL-10 (*p* = 0.0024), TNF (*p* = 0.0005), and IFN-*γ* (*p* = 0.0068), but not IL-17A. These data indicate that both ROS and cell-cell contact mediated through CD11b mediate the inhibition of all measured cytokines, except for IL-17A where cell-cell contact does not seem to play a role. Taken together, these data further suggest that NDGs do not promote the formation of Th2 and Th17 subsets and the loss of Th1, but argues for that the downregulation of CD183 might be due to internalization of the receptor or cleavage.

CD183, also known as CXCR3, has four known ligands, CXCL4 (PF4), CXCL9 (MIG), CXCL10 (IP-10), and CXCL11 (I-TAC) (17, 18). Upon ligand binding to CD183, CD183 is removed from the surface of T-cells in a rapid and dose-dependent manner (19). Internalized CD183 is degraded, and replenishment of the receptor occurs over several hours and requires de novo synthesis (19). To investigate if the loss of surface CD183 was dependent on binding of CXCL4, CXCL9, CXCL10, or CXCL11 to CD183, antibodies against these ligands were added to the culture. After 2 hours, the presence of CD183 on the surface of T-cells was evaluated using flow cytometry. As previously indicated, NDGs suppressed CD183 surface expression in T-cells. Blocking CXCL4, CXCL9, CXCL10, or CXCL11 did not protect CD183 expression on T-cells (Supplementary Figure 2). Several different concentrations (from 0.5 ug/ml to 50 ug/ml) were used, but none of them restored the level of CD183 on the surface of T-cells (data not shown). Our data indicate that NDG-induced suppression of CD183 is not mediated by ligand-binding to CD183.

## 4. Discussion

The regulation of the immune system and interaction between myeloid cells and T-cells in health and disease is complex. In this paper, we show that NDGs could exert MDSC activity and inhibit T-cell proliferation by cell-cell contact and ROS production. The inhibition is fast and can be observed within the first few hours of contact. We also show that NDGs suppress CD183 expression on the surface of T-cells, leading to an almost complete removal of the receptor from T-cell surface. Furthermore, NDGs generally suppress the production of Th-signature cytokines.

Several different mechanisms have been suggested for myeloid-derived suppression of T-cells, and our findings of suppression mediated by contact dependence through CD11b and production of ROS are in line with previous data from Aarts et. al (4); they also investigated cell-cell contact with cocultures with transwell technique and found that cell-cell contact was necessary for NGD-induced suppression of T-cells. However, it is possible that other mechanism could be important during diseases. In this study, we could not see any suppressive role of MMP9 activated TGF-*β*, a mechanism described by Germann et al. in neutrophils from patients with colon cancer (11).

CD11b is part of the integrin Mac-1 complex together with CD18 (4, 13). Blocking of CD11b prevent NDGs from inhibiting T-cell proliferation, indicating that cell-cell contact through the Mac-1 complex is necessary for the inhibitory effect. However, another study has claimed that CD11b only plays a minor role, and that upregulation of programmed death-ligand 1 (PD-L1) is more important for the inhibitory effect of neutrophils (12). We could not evaluate this mechanism since the antibodies we tried for blocking PD-L1 killed the T-cells (data not shown). Nonetheless, our data, together with data from above mentioned groups, show that CD11b does play a role and that cell-cell contact is important for the inhibitory effect.

Neutrophils are sensitive cells that easily become activated, and the purification process and the handling of the cells prior to the culture could potentially influence the outcome of these experiments. A few studies report that blood NDGs must be activated to exhibit MDSC function (4–8). However, we and other authors have observed inhibitory capacities without neutrophil activators (12, 21, 22). In our previous study, we showed that NDGs are not activated during the isolation process, but become activated approximately one hour after coincubation with activated T-cells, as measured by the surface expression of the neutrophil activation markers CD11b and CD66b (21). Interestingly, the loss of CD183 on the surface of the T-cells is more rapid than the activation of the neutrophils. In this study, we observed that the decrease of CD183 started within the first 30 minutes of coculture. The decline of T-cell activation markers occurred after 2 hours (21).

Here we show that NDGs did not have an inhibitory effect specifically on Th1 cells, but rather a broader inhibitory ability, as they downregulate production of all measured Th-cytokines in a dose-dependent manner. Neutrophils do not seem to drive Th response in a specific direction, but generally suppress T-cell cytokine production in all three Th subsets.

CD183 (CXCR3) is the receptor for four different chemokines, including CXCL4 (PF4), CXCL9 (MIG), CXCL10 (IP-10), and CXCL11 (I-TAC) (17, 18). There are two different isoforms of CD183, CXCR3-A and CXCR3-B. CXCL9, CXCL10, and CXCL11 bind to both CXCR3-A and CXCR3-B, while CXCL4 only bind the CXCR3-B isoform (17). CXCL9, CXCL10, and CXCL11 are all induced by IFN-*γ* and promote Th1 immune responses and Th1 migration (19) (20). A study by Romagnani et al. shows that CXCL10 promotes Th1 response and promotes production of IFN-*γ*, while CXCL4 promotes Th2 response by induction of Th2-type cytokines IL-4, IL-5, and IL-13 in naïve CD4^+^ T-cells (18). Furthermore, downregulation of CD183 has been linked to decreased migration in CD8^+^ T-cells in tumor patients (23). Since CXCR3 binding leads to internalization of the receptor and therefore removal from the surface of T-cells (19), we hypothesized that these chemokines might be responsible for the loss of CD183 on the surface of T-cells and perhaps played a role in promoting Th1 or Th2 responses. However, when adding blocking antibodies against the four chemokines, either alone or in combination, we could not revert the loss of the receptor. The loss of CD183 is not due to binding of its chemokine. NDGs only produce CXCL9, CXCL10, and CXCL11 after incubation with IFN-*γ* and an activator (24, 25). NDGs do, however, release extracellular content upon activation, including proteases (26). The proteases can cleave surface receptors, which could terminate cytokine responses (26). If the neutrophils release a protease that cleaves CD183 from the surface, it could possibly lead to the shutdown of at least the Th1 cytokine response. However, all Th cytokines were inhibited by neutrophils, indicating that removal of CD183 cannot be the only mechanism for cytokine inhibition.

NDGs suppressed all Th-type cytokines measured, including IL-2, IL-4, IL-6, IL-10, IL-17A, TNF, and IFN-*γ*. The different Th-type cytokines modulate the function of cells from both the adaptive and the innate immune system (27). Th cells are critical for the initiation of anti-tumor responses (28), and Th cells with reduced functional ability has been associated with the development of autoimmune diseases (27). The ROS inhibitor catalase could restore the cytokine production, indicating that ROS is part of the mechanism responsible for the suppression of these cytokines. The arginase inhibitor nor-NOHA did not protect cytokine production nor proliferative capacity. Therefore, arginase and nitric oxide does not seem to be important in our experimental set-up. When abrogating the close-contact between neutrophils and T-cells by blocking CD11b, the production of all cytokines, except IL-17, was partially restored. Close contact seems to be important for all the cytokines, except for IL-17. There are several different pathways acting together to create the inhibitory effect by neutrophils, and they still need to be further unraveled.

In this study, we showed that NDGs can interact with T-cells, and that this interaction led to inhibition of both proliferation and cytokine production. To suppress T-cells, NDGs produce ROS, but arginase and TGF-*β* were not part of these inhibitory mechanisms. The Th1 marker CD183 was rapidly downregulated from the surface of the T-cells after coincubation with NDGs, unrelated to the CD183 chemokine-binding. These data indicate that NDGs can regulate the adaptive immune response by inhibiting T-cell proliferation and cytokine production.

## Data Availability

Data is not accessible due to ethical reasons (patient privacy).

## Supplementary Materials

Supplementary Materials Supplementary Table 1. Study population characteristics. Supplementary Table 2. T-cell panel for flow cytometry. Supplementary Figure 1. Gating strategy for the flow cytometry analysis of T-cells over time. Supplementary Figure 2. Geometric mean of CD183 after 2 hours of coculture with CXCL4, CXCL9, CXCL10, CXCL11 or all of them in combination.
