# Supplementary material for: Suppression of T-Cell Proliferation by Normal Density Granulocytes Led to CD183 Downregulation and Cytokine Inhibition in T-Cells
Source: J Immunol Res. 2022 Nov 16;2022:8077281. doi: 10.1155/2022/8077281 (PMC9683987; doi:10.1155/2022/8077281)
Supplement: Supplementary Materials — Supplementary Table 1. Study population characteristics. Supplementary Table 2. T-cell panel for flow cytometry. Supplementary Figure 1. Gating strategy for the flow cytometry analysis of T-cells over time. Supplementary Figure 2. Geometric mean of CD183 after 2 hours of coculture with CXCL4, CXCL9, CXCL10, CXCL11 or all of them in combination. [file 8077281.f1.docx]

**Supplementary material**

**Table 1. Study population characteristics**

|  | Age (years) | Sex |
| --- | --- | --- |
| 1 | 44 | F |
| 2 | 47 | F |
| 3 | 48 | F |
| 4 | 43 | F |
| 5 | 56 | F |
| 6 | 55 | F |
| 7 | 45 | F |
| 8 | 51 | F |
| 9 | 36 | F |
| 10 | 72 | F |
| 11 | 41 | F |
| 12 | 37 | F |
| 13 | 53 | M |
| 14 | 39 | M |
| 15 | 27 | M |
| 16 | 28 | M |
| 17 | 43 | M |
| 18 | 47 | M |
| 19 | 34 | M |

**Table 2. T-cell panel for flow cytometry**

| Antibody against | Fluorescent marker | Clone | Company |
| --- | --- | --- | --- |
| CD3 | APC Cy7 | SK7 | BD Pharmingen |
| CD4 | BV421 | RPA-T4 | BD Horizon |
| CD8 | V500 | RPA-T8 | BD Horizon |
| CD25 | PerCP Cy5.5 | M-A251 | BD Pharmingen |
| CD57 | BV605 | QA17A04 | BioLegend |
| CD66b | AF700 | G10F5 | BioLegend |
| CD69 | PE Cy7 | FN50 | BD Pharmingen |
| CD127 | AF647 | HIL-7R-M21 | BD Pharmingen |
| CD183 | AF488 | G025H7 | BioLegend |
| CD196 | PE | G034E3 | BioLegend |
| CD279 | CF594 | EH12.1 | BD Horizon |
| TCRγδ | BV650 | B1 | BD Horizon |

**Supplementary figure 1. Gating strategy for the flow cytometry analysis of T-cells over time.** Lymphocytes were gated from single cells, whereupon the CD3^+^ cells were gated. From the CD3^+^ cells, CD4^+^ helper T-cells and CD8+ T-cells were gated. Th1, Th2 and Th17 cells were gated from the CD4^+^ population based on their expression of CD196 (CCR6) and CD183 (CXCR3).

**Supplementary figure 2. Geometric mean of CD183 after 2 hours of co-culture with CXCL4, CXCL9, CXCL10, CXCL11 or all of them in combination**. Graph indicate median with 95% CI (n=3).
